# Ascending aortic replacement versus aortic root replacement in patients with type A aortic dissection involving the aortic root

**DOI:** 10.1177/17539447241303408

**Published:** 2025-01-22

**Authors:** Fausto Biancari, Giuseppe Gatti, Timo Mäkikallio, Tatu Juvonen, Giovanni Mariscalco, Zein El-Dean, Matteo Pettinari, Javier Rodriguez Lega, Andrea Perrotti, Francesco Onorati, Konrad Wisniewki, Till Demal, Petr Kacer, Dario Di Perna, Igor Vendramin, Mauro Rinaldi, Luisa Ferrante, Eduard Quintana, Joscha Buech, Caroline Radner, Antonio Fiore, Angelo M. Dell’Aquila, Paola D’Errigo, Stefano Rosato, Gianluca Polvani, Sven Peterss

**Affiliations:** Department of Cardiovascular Surgery, Centro Cardiologico Monzino IRCCS, Via Carlo Parea 4, Milan 20138, Italy; Division of Cardiac Surgery, Cardio-thoracic and Vascular Department, Azienda Sanitaria Universitaria Giuliano Isontina, Trieste, Italy; Department of Medicine, South-Karelia Central Hospital, University of Helsinki, Lappeenranta, Finland; Heart and Lung Center, Helsinki University Hospital, University of Helsinki, Helsinki, Finland; Faculty of Medicine, University of Oulu, Oulu, Finland; Department of Cardiac Surgery, Glenfield Hospital, Leicester, UK; Department of Cardiac Surgery, Glenfield Hospital, Leicester, UK; Department of Cardiac Surgery, Ziekenhuis Oost-Limburg, Genk, Belgium; Cardiovascular Surgery Department, University Hospital Gregorio Marañón, Madrid, Spain; Department of Thoracic and Cardiovascular Surgery, University of Franche-Comte, Besancon, France; Division of Cardiac Surgery, University of Verona Medical School, Verona, Italy; Department of Cardiothoracic Surgery, University Hospital Muenster, Muenster, Germany; Department of Cardiovascular Surgery, University Heart and Vascular Center Hamburg, Hamburg, Germany; Department of Cardiac Surgery, Charles University and University Hospital Kralovske Vinohrady, Prague, Czech Republic; Department of Cardiac Surgery, Centre Hospitalier Annecy Genevois, Epagny Metz-Tessy, France; Cardiothoracic Department, Azienda Sanitaria Universitaria Friuli Centrale, Udine, Italy; Cardiac Surgery, Molinette Hospital, University of Turin, Turin, Italy; Cardiac Surgery, Molinette Hospital, University of Turin, Turin, Italy; Department of Cardiovascular Surgery, Hospital Clínic de Barcelona, University of Barcelona, Barcelona, Spain; Department of Cardiac Surgery, LMU University Hospital, Ludwig Maximilian University, Munich, Germany; German Centre for Cardiovascular Research, Partner Site Munich Heart Alliance, Munich, Germany; Department of Cardiac Surgery, LMU University Hospital, Ludwig Maximilian University, Munich, Germany; German Centre for Cardiovascular Research, Partner Site Munich Heart Alliance, Munich, Germany; Department of Cardiac Surgery, Hôpitaux Universitaires Henri Mondor, Assistance Publique-Hôpitaux de Paris, Creteil, France; Department of Cardiothoracic Surgery, University Hospital Muenster, Muenster, Germany; Department of Cardiac Surgery, Martin Luther University Halle-Wittenberg, Halle, Germany; National Center for Global Health, Istituto Superiore di Sanitá, Rome, Italy; National Center for Global Health, Istituto Superiore di Sanitá, Rome, Italy; Department of Cardiovascular Surgery, Centro Cardiologico Monzino IRCCS, Milan, Italy; Department of Biomedical, Surgical and Dental Sciences, University of Milan, Milan, Italy; Department of Cardiac Surgery, LMU University Hospital, Ludwig Maximilian University, Munich, Germany

**Keywords:** aortic dissection, aortic root, Bentall procedure, David procedure, reoperation, type A aortic dissection

## Abstract

**Background:**

Extensive surgical resection of the thoracic aorta in patients with type A aortic dissection (TAAD) is thought to reduce the risk of late aortic wall degeneration and the need for repeat aortic operations.

**Objectives:**

We evaluated the early and late outcomes after aortic root replacement and supracoronary ascending aortic replacement in patients with TAAD involving the aortic root.

**Design:**

Retrospective, multicenter cohort study.

**Methods:**

The outcomes after aortic root replacement and supracoronary ascending aortic replacement in patients with TAAD involving the aortic root, that is dissection flap located at least in one of the Valsava segments, were herein evaluated. In-hospital mortality, neurological complications, dialysis as well as 10-year repeat proximal aortic operation, and mortality were the outcomes of this study.

**Results:**

Supracoronary ascending aortic replacement was performed in 198 patients and aortic root replacement in 215 patients. During a mean follow-up of 4.0 ± 4.0 years, 19 patients underwent 22 repeat procedures on the aortic root and/or aortic valve. No operative death occurred after these reinterventions. The risk of proximal aortic reoperation was significantly lower in patients who underwent aortic root replacement (5.5% vs 12.9%, adjusted subdistributional hazard ratio (SHR) 0.085, 95% CI 0.022–0.329). Aortic root replacement was associated with higher rates of in-hospital (14.4% vs 12.1%, adjusted odds ratio 2.192, 95% CI 1.000–4.807) and 10-year mortality (44.5% vs 30.4%, adjusted hazard ratio 2.216, 95% CI 1.338–3.671). Postoperative neurological complications and dialysis rates were comparable in the study groups.

**Conclusion:**

Among patients with TAAD involving the aortic root, its replacement was associated with a significantly lower rate of repeat proximal aortic operation of any type compared to supracoronary aortic replacement. Still, aortic root replacement seems to be associated with an increased risk of mortality in these patients.

**Trial registration:**

ClinicalTrials.gov: NCT04831073 (https://clinicaltrials.gov/study/NCT04831073).

## Introduction

Acute type A aortic dissection (TAAD) is a severe condition that requires prompt surgical repair. Extensive surgical resection of the thoracic aorta in patients with TAAD is thought to reduce the risk of late aortic wall degeneration and the need for repeat aortic operations.^1–3^ However, multicenter studies failed to demonstrate reduced rates of aortic reoperations after extensive repair of the thoracic aorta compared to surgical repair limited to the ascending aorta.^4–6^ Still, we recognize that subsets of patients with TAAD may benefit from an extensive aortic repair. Aortic dissection involving the aortic root may potentially pose patients at risk of aortic wall degeneration and/or significant dysfunction of the aortic valve. Data on the outcome of TAAD involving the aortic root are lacking and surgeons face the dilemma of whether to adopt an aggressive surgical strategy in these patients.^7,8^ In the present study, we evaluated the early and late outcomes of patients with acute TAAD involving one or more sinuses of Valsalva who underwent supracoronary replacement of the ascending aorta or aortic root replacement from a multicenter study.

## Materials and methods

### Study population

This study is an analysis of an observational study of a cohort of patients from the European Registry of Type A Aortic Dissection (ERTAAD), which was a multicenter study of a retrospective nature including 3735 consecutive patients operated for acute TAAD at 17 centers of cardiac surgery in eight European countries (Belgium, Czech Republic, Finland, France, Germany, Italy, Spain, and the United Kingdom) from January 2005 to March 2021. The Ethical Review Board of the Helsinki University Central Hospital, Finland (April 21, 2021, diary no. HUS/237/2021) and of each participating hospital approved this study. The patient’s informed consent was waived because of the retrospective nature of the study. Data were collected into a Microsoft Access datasheet (Redmond, WA, USA) with pre-specified variables. Data on late mortality and aortic reoperations were gathered from national registries as well as by contacting regional hospitals, patients, and their relatives.

**Figure 1.**
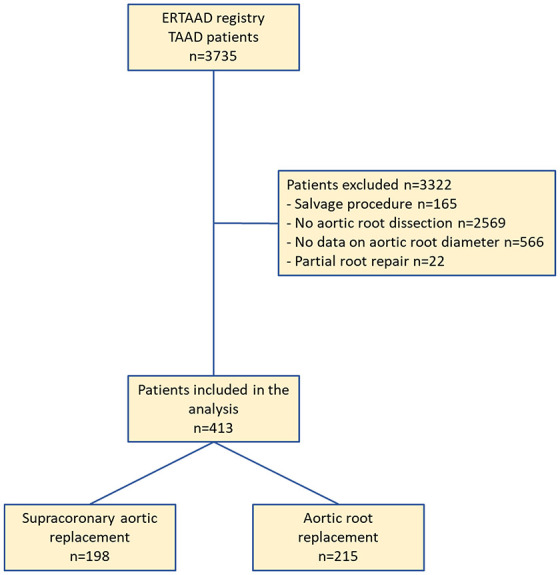
Study flow chart.

Patients with aortic dissection involving the aortic root, that is dissection flap located at least in one of the Valsava segments, were the subjects of this study. The exclusion criteria of this analysis were the following: salvage procedure, no aortic root dissection, lack of data on the aortic root diameter, and partial root surgical repair. Patients with supracoronary aortic replacement with partial resection and replacement of the dissected sinuses of Valsalva were excluded from the analysis because of their limited number (Figure 1). The outcomes of patients who underwent ascending aortic replacement were compared with those of patients who underwent aortic root replacement, that is the Bentall procedure as well as the David and the Yacoub procedures.

### Outcomes

The primary outcome of this study was proximal aortic reoperation at 10 years. Proximal aortic reoperation refers to any surgical reoperation on the aortic root and/or aortic valve. Secondary outcomes were death from any cause occurring during the index hospitalization, that is in-hospital mortality, any postoperative neurological event that occurred during the index hospitalization defined as ischemic stroke, hemorrhagic stroke and/or global brain ischemia, renal replacement therapy, and all-cause death at 10 years.

### Statistical analysis

Sample size calculation was not performed for this study because there are no data available on the outcome of patients with TAAD involving the aortic root. Continuous variables were reported as the means and standard deviations, while categorical variables were reported as counts and percentages. Missing data were not replaced in the analyses. Univariate analysis was performed using the Mann–Whitney test, Chi-square test, and Fisher’s exact test. Survival analysis was performed using Kaplan–Meier’s test and cumulative incidences of repeat aortic operations were calculated using the competing risk analysis with Gray’s test considering mortality as the competing event. We did not perform propensity score matching analysis because of the relatively limited size of the study cohorts. Multilevel mixed-effects logistic regression was used to estimate the adjusted risk of in-hospital mortality, neurological complications, and dialysis considering the cluster effect of participating hospitals. Multilevel mixed-effects parametric survival regression was used to estimate the risk of late mortality. The results of the competing risk analysis were also adjusted for the participating hospitals. All regression analyses were adjusted for the following covariates with *p* < 0.2 in univariate analysis: age, gender, aortic root diameter, severity of aortic valve insufficiency, number of dissected Valsava sinuses, bicuspid aortic valve, prior cardiac surgery, iatrogenic TAAD, pulmonary disease, extracardiac arteriopathy, renal malperfusion, mesenteric malperfusion, peripheral malperfusion, preoperative invasive mechanical ventilation, aortic arch dissection, and concomitant coronary artery bypass grafting. The partial or total aortic arch replacement was forced into these regression models. Risk estimates were reported as odds ratios (ORs), hazard ratios (HRs), and subdistributional hazard ratios (SHRs) with their 95% confidence intervals (CIs). Statistical significance was set at *p* < 0.05. Statistical analyses were performed with Stata statistical software (version 15.1, StataCorp LLC, College Station, TX, USA).

## Results

The ERTAAD dataset included data from 3735 consecutive patients who underwent surgery for TAAD. The study flowchart (Figure 1) summarizes the number of patients excluded from this analysis with reason. The present study included 413 patients fulfilling the inclusion and exclusion criteria of this analysis. Supracoronary aortic replacement was performed in 198 patients and aortic root replacement, that is the Bentall procedure, the David procedure, or the Yacoub procedure, was performed in 215 patients (Figure 1). Clinical characteristics and operative data of these patients are summarized in Table 1.

**Table 1.** Baseline characteristics, operative variables, and outcomes of patients who underwent supracoronary aortic replacement or aortic root replacement.

Variables	Supracoronary aortic replacement, *N* = 198	Aortic root replacement, *N* = 215	*p* Values	Multivariable adjusted risk estimates, 95% CI
Baseline variables				
Age, years	63.2 (11.9)	58.5 (13.5)	<0.001	
Females	66 (33.3)	45 (20.9)	0.005	
Onset of symptoms to surgery, h	16 (23)	18 (28)	0.476	
Aortic root diameter, mm	41 (8)	47 (11)	<0.001	
No. of dissected Valsalva sinuses			<0.001	
1	115 (58.1)	95 (44.2)		
2	67 (33.8)	48 (22.3)		
3	16 (8.1)	72 (33.5)		
Aortic valve insufficiency			<0.001	
No/trace	57 (29.2)	34 (15.8)		
Mild	58 (29.7)	41 (19.1)		
Moderate	35 (18.0)	58 (27.0)		
Severe	45 (23.1)	82 (38.1)		
Bicuspid aortic valve	4 (2.0)	25 (11.6)	<0.001	
Genetic syndrome	6 (3.0)	8 (3.7)	0.698	
Familial history of aortic dissection/aneurysm	10 (5.1)	12 (5.6)	0.810	
Prior cardiac surgery	8 (4.0)	3 (1.4)	0.128	
Iatrogenic dissection	9 (4.5)	4 (1.9)	0.160	
Diabetes	12 (6.1)	18 (8.4)	0.386	
Prior stroke	10 (5.1)	11 (5.1)	0.976	
Pulmonary disease	15 (7.6)	9 (4.2)	0.141	
Extracardiac arteriopathy	12 (6.1)	2 (0.9)	0.005	
Preoperative malperfusion				
Cerebral malperfusion	36 (18.2)	38 (17.7)	0.893	
Spinal malperfusion	7 (3.5)	3 (1.4)	0.206	
Renal malperfusion	37 (18.7)	12 (5.6)	<0.001	
Mesenteric malperfusion	11 (5.6)	5 (2.3)	0.125	
Peripheral malperfusion	46 (23.2)	37 (17.2)	0.127	
Cardiogenic shock requiring inotropes	31 (15.7)	26 (12.1)	0.294	
Invasive mechanical ventilation	20 (10.1)	10 (4.7)	0.033	
Aortic arch dissection	177 (89.4)	178 (82.8)	0.054	
Operative variables				
Tear in the aortic root	51 (25.8)	98 (45.6)	<0.001	
Bentall procedure	—	180 (83.7)	—	
David procedure	—	29 (13.5)	—	
Yacoub procedure	—	6 (2.8)	—	
Aortic valve replacement	20 (10.1)	—	—	
Aortic valve repair	3 (1.5)	2 (0.9)	0.587	
Anastomosis reinforced with Teflon/pericardium strip	137 (70.3)	74 (34.4)	<0.001	
Use of bovine serum albumin and glutaraldehyde glue	152 (76.8)	96 (44.7)	<0.001	
Partial/total arch replacement	47 (23.7)	42 (19.5)	0.299	
CABG	18 (9.1)	40 (18.6)	0.005	
CABG because of coronary ostia injury	9 (4.5)	27 (12.6)	0.004	
Aortic cross-clamping time, min	110 (56)	167 (58)	<0.001	
Cardiopulmonary bypass time, min	206 (78)	260 (90)	<0.001	
Outcomes				
In-hospital mortality	24 (12.1)	31 (14.4)	0.492	2.192, 1.000–4.807
Stroke/global brain ischemia	45 (20.2)	27 (17.2)	0.160	0.706, 0.360–1.386
Dialysis	40 (20.2)	27 (12.6)	0.035	0.573, 0.283–1.159
10-year mortality	50 (30.4)	64 (44.5)	0.603	2.216, 1.338–3.671
10-year proximal aortic reoperation	13 (12.9)	6 (5.5)	0.040	0.086, 0.022–0.329

Continuous data are reported as mean and standard deviation (in parentheses). Categorical data are reported as counts and percentages (in parentheses). Risk estimates are odds ratios, hazard ratios, and subdistributional hazard ratios with 95% CI.

CABG, coronary artery bypass grafting; CI, confidence interval.

Patients who underwent aortic root replacement were significantly younger and had a higher prevalence of bicuspid aortic valve, severe aortic valve insufficiency, and TAAD involving all three Valsalva sinuses compared to patients who underwent supracoronary ascending aortic replacement (Table 1). Concomitant coronary artery bypass grafting was required more frequently during aortic root replacement than supracoronary aortic replacement, mostly because of coronary ostia injury (*p* = 0.004). Aortic root replacement required markedly longer periods of myocardial ischemia (mean, 167 ± 58 min vs 110 ± 56 min, *p* < 0.001) and of cardiopulmonary bypass (mean, 260 ± 90 min vs 206 ± 78 min, *p* < 0.001).

During a mean follow-up of 4.0 ± 4.0 years, 19 patients underwent 22 repeat procedures on the aortic root. Nine procedures were performed in 6 patients after aortic root replacement and 13 procedures were performed in 13 patients after ascending aortic replacement. Data on these procedures and their related indications are summarized in Table 2. No operative death occurred after these reoperations.

**Table 2.** Repeat procedures on the aortic root and aortic valve and their related indications.

Patients	Index surgical procedure	No. reoperations	Indication for reoperation	Repeat procedures on the aortic root/aortic valve
1	Bentall procedure	4	Pseudoaneurysm of the right coronary button, pseudoaneurysm of the proximal anastomosis of the vein graft, prosthesis infection	Local repair twice and CABG, Amplatzer plug insertion, Bentall procedure
2	Bentall procedure	1	Pseudoaneurysm of the right coronary button	Local repair
3	Bentall procedure	1	Prosthesis infection	Bentall procedure
4	Bentall procedure	1	Prosthesis infection	Bentall procedure
5	Bentall procedure	1	Pseudoaneurysm of the proximal anastomosis	Local repair
6	David procedure	1	Aortic valve insufficiency	SAVR
7	Supracoronary aortic replacement	1	Pseudoaneurysm of the aortic root	Bentall procedure
8	Supracoronary aortic replacement	1	Unknown	Yacoub procedure
9	Supracoronary aortic replacement	1	Aortic valve insufficiency	SAVR
10	Supracoronary aortic replacement	1	Aortic valve insufficiency	TAVR
11	Supracoronary aortic replacement	1	Aneurysm	Bentall procedure
12	Supracoronary aortic replacement	1	Pseudoaneurysm of the aortic root	Bentall procedure
13	Supracoronary aortic replacement	1	Prosthesis infection	Supracoronary aortic repair
14	Supracoronary aortic replacement	1	Aortic valve insufficiency	SAVR
15	Supracoronary aortic replacement	1	Pseudoaneurysm of the aortic root, aortic valve insufficiency	SAVR, supracoronary aortic repair
16	Supracoronary aortic replacement	1	Pseudoaneurysm of the proximal anastomosis	SAVR, supracoronary aortic repair
17	Supracoronary aortic replacement	1	Pseudoaneurysm of the proximal anastomosis	Bentall procedure
18	Supracoronary aortic replacement	1	Pseudoaneurysm of the proximal anastomosis	Supracoronary aortic repair
19	Supracoronary aortic replacement	1	Pseudoaneurysm of the proximal anastomosis	SAVR, supracoronary aortic repair

CABG, coronary artery bypass grafting; SAVR, surgical aortic valve replacement; TAVR, transcatheter aortic valve replacement.

**Figure 2.**
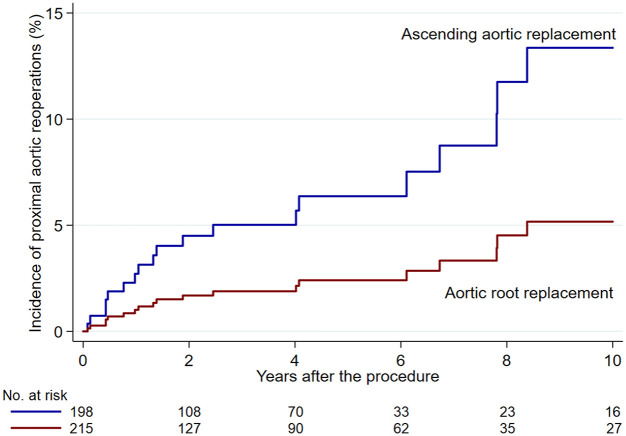
Cumulative incidence of repeat proximal aortic procedures in patients who underwent aortic root replacement or supracoronary aortic replacement (adjusted SHR 0.085, 95% CI 0.022–0.329). SHR, subdistributional hazard ratio.

The risk of proximal aortic reoperation was significantly lower in patients who underwent aortic root replacement (crude cumulative incidence rates 5.5% vs 12.9%, adjusted SHR 0.085, 95% CI 0.022–0.329, *p* < 0.001; Figure 2). The number of sinuses of Valsalva involved by aortic dissection was not associated with an increased risk of repeat proximal aortic reoperation (adjusted SHR 1.078, 95% CI 0.365–3.182). Tissue glue applied to the dissected aortic layers did not prevent proximal aortic reoperations (adjusted SHR 1.397, 95% CI 0.244–7.984). Among patients with tear of any extent of the aortic root, aortic root replacement reduced significantly the risk of proximal aortic reoperation (adjusted SHR 0.078, 95% CI 0.009–0.648) compared to ascending aortic replacement.

Aortic root replacement was associated with a significantly higher risk of in-hospital (crude rates 14.4% vs 12.1%, adjusted OR 2.192, 95% CI 1.000–4.807, *p* = 0.050; likelihood ratio test, *p* < 0.001) and 10-year mortality (crude rates 44.5% vs 30.4%, adjusted HR 2.216, 95% CI 1.338–3.671, *p* = 0.002; likelihood ratio test, *p* < 0.001). The rates of in-hospital mortality were comparable after aortic root replacement and ascending aortic replacement in patients with tears located in the aortic root (8.2% vs 11.8%, respectively, *p* = 0.557). The higher risk of 10-year mortality after aortic root replacement was statistically significant also after excluding from the analysis those patients who died during the index hospitalization (crude rates 35.1% vs 20.1%, adjusted HR 2.125, 95% CI 1.015–4.447, *p* = 0.045; likelihood ratio test, *p* < 0.001). The rates of postoperative neurological complications and dialysis were not significantly different between the study groups (Table 1).

## Discussion

The main findings of this study investigating patients with TAAD involving the aortic root can be summarized as follows: (1) surgical replacement of the aortic root was associated with a significantly lower rate of repeat proximal aortic operation of any type compared to supracoronary ascending aortic replacement; (2) aortic root replacement was associated with an increased risk of early and late mortality in these patients. These findings should be evaluated considering significant differences in the risk profile of patients treated with sparing or replacement of the dissected aortic root. In fact, patients who underwent supracoronary replacement of the ascending aorta were significantly older than those who underwent aortic root replacement and more frequently were admitted to the operating room with invasive mechanical ventilation (Table 1). On the other hand, the decision to perform aortic root replacement instead of supracoronary aortic replacement was supported by a larger diameter of the aortic root and a higher prevalence of aortic valve insufficiency (Table 1). Still, we may expect that several patients underwent aortic root replacement due to the surgeon’s policy of achieving complete resection of the dissected aorta. In fact, less than severe aortic valve regurgitation was present in two-thirds of patients who underwent aortic root replacement, and the diameter of the aortic root was not excessively large in all these patients (mean diameter, 47 ± 11 mm). Still, we cannot exclude that in several patients, extensive surgical repair was justified by severe injury or frailty of the dissected aortic wall, which made the procedure more complex and might have contributed to increased early mortality. Taken together, these findings suggest that the study cohorts differed in terms of risk profile and anatomic characteristics of the dissected aorta. However, when risk estimates of early and late outcomes were adjusted for multiple baseline risk factors and operative variables, aortic root replacement was associated with a significantly lower incidence of repeat proximal aortic operations. This was particularly evident when the tear involved the aortic root. Early and late mortality rates were significantly higher than in patients who underwent an aortic root-sparing procedure. However, the negative prognostic effect on 10-year mortality was not only due to the increased in-hospital mortality rate among patients who underwent aortic root replacement. It is worth considering that the adjusted CI of the risk estimate of in-hospital mortality was rather large and its lower limit was close to 1.0. This observation suggests that a type II error might introduce bias in the present analysis and the negative prognostic impact of aortic root replacement was of limited statistical significance. However, a higher risk of mortality could have been expected in these patients due to the complexity of the procedure and the markedly longer periods of myocardial ischemia and cardiopulmonary bypass required to accomplish surgical replacement of the aortic root (Table 1). Therefore, when feasible, a policy of limited aortic resection can be advocated in high-risk patients. Noteworthy, the cumulative incidence of proximal aortic reoperation at 10 years due to such late complications was not excessive after supracoronary ascending aortic replacement, and repeat aortic procedures were not associated with operative mortality. Peterss et al.^9^ demonstrated that sparing the aortic root was associated with durable results compared to aortic root replacement. However, the development of pseudoaneurysm at the level of the proximal suture line is not uncommon after supracoronary aortic replacement^10^ as confirmed in the present series. On the other hand, these findings suggest that aortic root replacement can be indicated in low-risk patients with long-life expectancy, still considering that the risk of complications indicating proximal aortic reoperation was not nihil also in these patients.

The main limitation of this analysis resides in the retrospective nature of this study. Second, the lack of data on the diameter of the aortic root in a rather large number of patients reduced the sample size of this series. However, we believe that knowledge of the size of the aortic root before surgery is crucial for an adequate adjustment of the risk estimates for reoperation on the aortic root and aortic valve. Post hoc sample size estimation (alpha 0.050, power 0.80) based on the cumulative incidences of proximal aortic reoperation at 10 years showed that 134 patients per group would have been enough to reject the null hypothesis. Therefore, the sample size was likely enough large to evaluate the primary outcome of this analysis. However, the sample size was not enough to reject the null hypothesis of a significant difference in terms of in-hospital mortality between the study groups, because such an analysis would have required 3410 patients in each study group (alpha 0.050, power 0.80). Furthermore, the adjusted CI of the risk estimate of in-hospital mortality was rather large and its lower limit was close to 1.0. Third, the limited number of patients who underwent replacement of the ascending aorta associated with partial resection of dissected Valsalva sinus/sinuses prevented an analysis of whether a limited surgical repair of the aortic root might suffice to reduce the risk of degeneration of the aortic root wall and aortic valve insufficiency. Fourth, we do not have data on whether the aortic valve was surgically resuspended. This is a significant limitation of the study because aortic valve resuspension may restore the structural integrity of the aortic annulus.^11^ Fifth, the individual surgeon’s decision to replace the aortic root certainly might have been dictated by the diameter of the aortic root and the status of the aortic wall as well as the severity of aortic valve insufficiency. However, the present data and clinical experience suggest that supracoronary aortic replacement is technically feasible in many patients with TAAD involving the aortic root. Hence, the extent of surgical repair can be influenced by the individual surgeon’s experience in aortic surgery and her/his strategy of TAAD repair. Sixth, these results can be biased by differences in the volume and specific experience in aortic surgery. We adjusted the results of this study considering the cluster effect of the participating hospitals, but the limited number of patients prevented an analysis of the impact of the individual surgeon on the outcomes. Finally, the prevalence of aortic dissection involving the aortic root might have been underreported. This might be related to the retrospective nature of the study. However, the reported data dealt with the extent of dissection into different segments of the aortic root. This information was likely reliable because the operating surgeons had to substantiate the decision to perform an aortic root replacement instead of a replacement of the ascending aorta. Therefore, we may expect that, even in the case of an underreporting of aortic root dissection, the data on the subjects included in this analysis were sufficiently detailed and reliable.

The strengths of this analysis are the multicenter nature of this study, and all patients were treated for TAAD involving the aortic root. Therefore, these results can be generalizable to other settings and may provide insights into the treatment of this specific condition.

In conclusion, the results of this study demonstrated that among patients with TAAD involving the aortic root, its replacement was associated with a significantly lower rate of repeat proximal aortic operation of any type compared to supracoronary ascending aortic replacement. Aortic root replacement was associated with an increased risk of early and late mortality in these patients. Noteworthy, we could not exclude that severe injury of the aortic root indicated its replacement, and this might have contributed to a higher rate of mortality. Still, the present findings suggest that, when feasible, surgical treatment of TAAD involving the aortic root could be tailored according to the risk profile of the patient. Indeed, the risk of proximal aortic reoperation at 10 years was not excessive after supracoronary ascending aortic replacement, and repeat procedures were not associated with operative mortality. Therefore, a policy of limited aortic resection can be advocated in high-risk patients, while aortic root replacement can be indicated in low-risk patients with long life expectancy. Further studies should explore the outcomes of patients who underwent partial surgical repair of the aortic root involved by TAAD.

## Declarations

**Ethics approval and consent to participate:** The Ethical Review Board of the Helsinki University Central Hospital, Finland (April 21, 2021, diary no. HUS/237/2021) and that of each participating hospital approved this study.

**Consent for publication:** Not applicable.

**Availability of data and materials:** The data of this study are not publicly available due to privacy issues.
